# Beyond the Bottle: Niacin Deficiency and Chronic Alcoholism

**DOI:** 10.7759/cureus.49482

**Published:** 2023-11-27

**Authors:** Ariba Ahmed, Sourya Acharya, Samarth Shukla, Ateeba Ahmed, Vijay Ravikumar

**Affiliations:** 1 Medical Education, Jawaharlal Nehru Medical College, Datta Meghe Institute of Higher Education and Research, Wardha, IND; 2 Medicine, Jawaharlal Nehru Medical College, Datta Meghe Institute of Higher Education and Research, Wardha, IND; 3 Pathology, Jawaharlal Nehru Medical College, Datta Meghe Institute of Higher Education and Research, Wardha, IND; 4 Psychiatry, Jawaharlal Nehru Medical College, Datta Meghe Institute of Higher Education and Research, Wardha, IND

**Keywords:** pellagra, alcohol, lesions, tryptophan, niacin

## Abstract

Vitamin B3 is called niacin, an essential nutrient for the human body. In diet, it exists in three forms - niacin, nicotinamide, and nicotinamide riboside and can also be produced from an amino acid - tryptophan in the gut. During the digestive process, these dietary forms of vitamin B3 get converted into nicotinamide adenine dinucleotide (NAD), which behaves as a cofactor and substrate in critical cellular reactions and thus plays a pivotal role in energy metabolism. The deficiency of this particular vitamin in the body, which manifests in different ways, is called Pellagra. We discuss one such case of niacin deficiency presenting with multisystem involvement in a chronic alcoholic.

## Introduction

A deficiency of niacin and its precursor tryptophan leads to the development of  pellagra - a nutritional deficiency disorder with multisystem involvement. The term pellagra comes from two Italian words “pellet” for skin and “agra” for rough, describing the skin’s characteristic roughened and coarse appearance in pellagrics. Don Gaspar Casal, a Spanish physician, was the first to identify and bring to notice pellagra among Spanish peasants in 1735. He called it “mal de la rosa” - the disease of red rash. However, it was often misdiagnosed as leprosy. Many names were given to this disease, such as “Asturian leprosy” or “Italian leprosy,” “Lombardy leprosy,” “rose disease,” “Alpine scurvy,” “Saint-Aman's disease” or “Stracham’s disease” before the name pellagra was coined by Frapolli in 1771.

Several decades later, in the beginning of the 20th century, the cause of pellagra was discovered by Dr. Joseph Goldberger, a renowned American physician and Epidemiologist [1]. Pellagra is often called “the disease of four D’s” - dermatitis, diarrhea, dementia, and death. Niacin, also called pellagra preventing factor, acts as a precursor to the coenzymes nicotinamide adenine dinucleotide (NAD) and NAD phosphate (NADP). These coenzymes play a crucial role as electron carriers in the oxidation-reduction reactions at the cellular level. For this reason, the tissues with high turnover rates like the gastrointestinal tract and skin, or tissues with high energy requirements like the brain, are affected first when niacin is deficient [2].

While the most common cause of pellagra is a dietary deficiency of niacin and tryptophan, it may also be seen in association with malnutrition, liver cirrhosis, anorexia nervosa, AIDS, Hartnup’s disease, and consumption of drugs like azathioprine, carbidopa, isoniazid, and chloramphenicol [3]. It is common  in developing countries especially where maize and jowar are the staple food. This deficiency disorder, though easy to treat, can easily be missed and thus requires a great degree of surmise and clinical knowledge in a physician [4]. We report the case of a middle-aged man who reported to a tertiary care hospital in rural part of central India in a state of irritability and a history of abstinence from alcohol.

## Case presentation

A 53-year-old male patient was brought to the emergency department in a condition of irritability and loss of behavioral control following a history of alcohol withdrawal for three days, as told by the accompanying relative. The Clinical Institute Withdrawal Assessment-Alcohol revised (CIWA- Ar) score of the patient was 4 indicating mild alcohol withdrawal syndrome (AWS) (Table 1) [5].

**Table 1:** CIWA-Ar score of the patient for assessment of AWS

Symptoms	Score
Nausea or vomiting	0
Tremor	0
Paroxysmal sweats	0
Anxiety	1
Agitation	3
Tactile disturbances	0
Auditory disturbances	0
Visual disturbances	0
Headache	0
Orientation/clouding of sensorium	0

**Figure 1:**
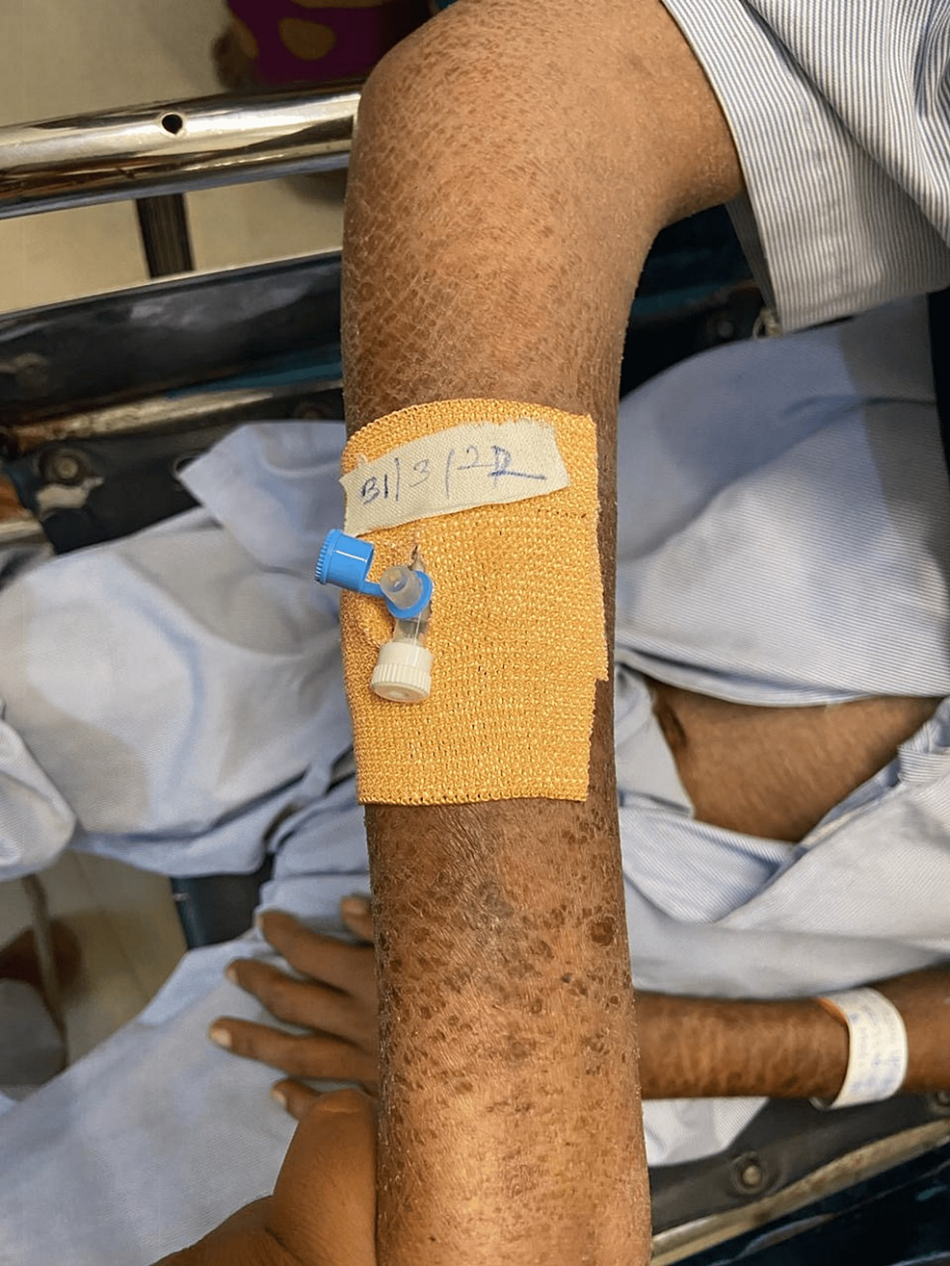
Skin lesions over the extensor aspect of right forearm

**Figure 2:**
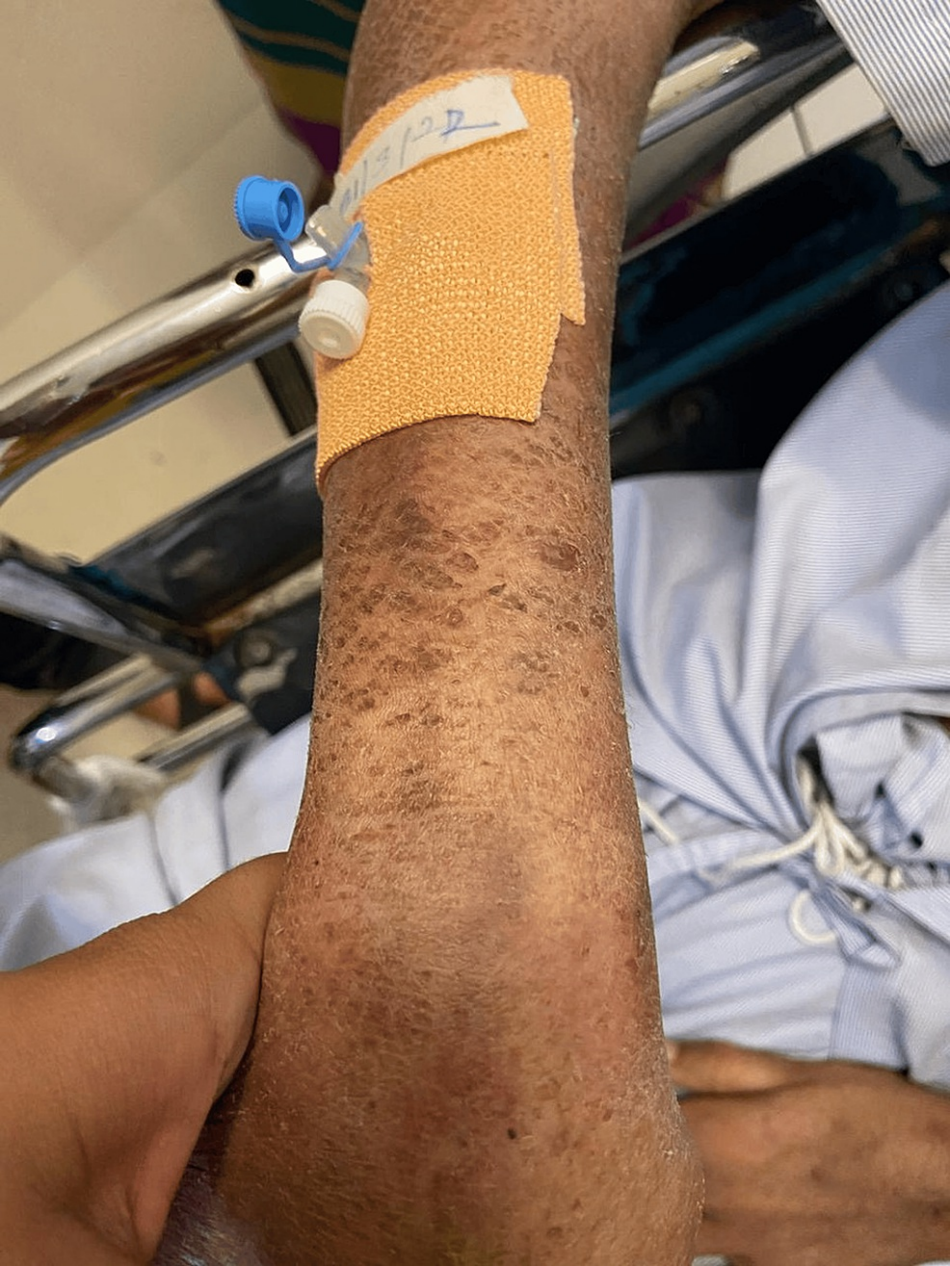
Skin lesions over distal part of extensor aspect of right forearm

**Figure 3:**
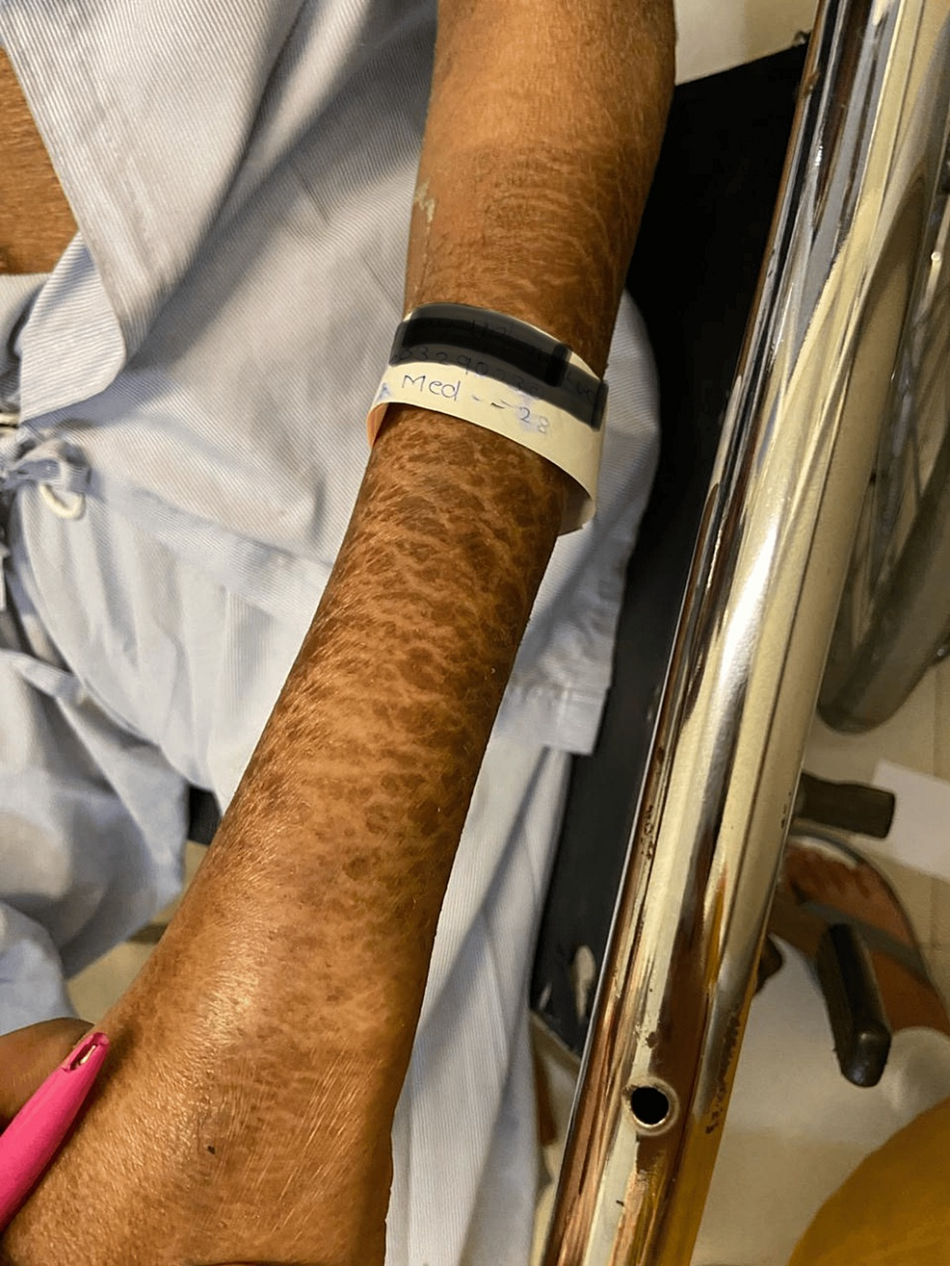
Skin lesions seen over the extensor aspect of left forearm

**Figure 4:**
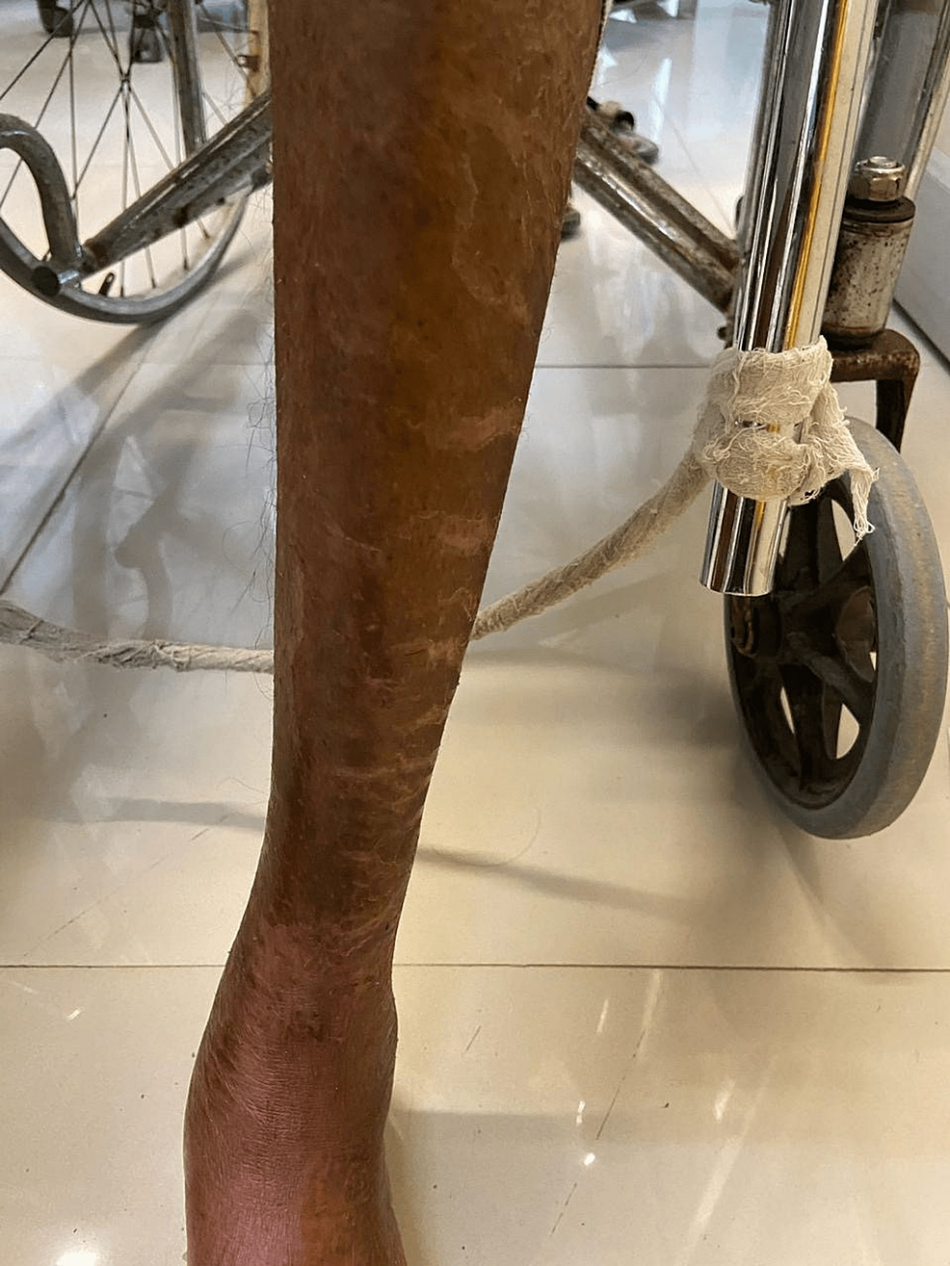
Anterior part of left leg shows rough, hyperpigmented skin with fissures The lesions are non-circumscr

The patient has been an alcohol addict for 25 years and has failed numerous attempts to give up the addiction. As informed by the relative, he has also been suffering from recurrent diarrhea episodes for the last three months. Hypoglycemia was ruled out by performing a bedside glucose measurement test. The patient was then administered lorazepam (4mg/mL of solution intravenously). The patient was conscious and irritable on examination, with preserved reflexes. Pulse was 90 beats per minute, regular in rhythm and volume, with no unique character. Blood pressure was 142/88 mm of Hg, taken in the right arm with the patient in the supine position. Pallor and Icterus were present bilaterally on the lower palpebral and upper bulbar conjunctiva respectively. Dry, cracked, hyperpigmented skin lesions were seen bilaterally on the upper limbs as well as lower limbs, particularly in the sun-exposed areas (Figures 1-4).

The abdominal examination revealed tender hepatomegaly and an increase in the frequency of bowel sounds. Heart sounds were normal with no murmur, bilaterally symmetrical chest rise with no crepitations or cough. Mental status examination found that the patient was conscious but irritable and non-compliant. He was unable to understand basic instructions and had a slow response. Relatives revealed history of several instances of the patient cursing the family members for no obvious reason and manifesting inappropriate emotions. Patient scored 18 in the Mini Mental Status Examination (MMSE)- indicating moderate impairment of cognition (Table 2) [6].

**Table 2:** Mini Mental Status Examination score table

Domain	Maximum score	Patient's score
Orientation	10	7
Registration	3	2
Attention and Calculation	5	2
Recall	3	2
Language test	2	2
Repetition	1	1
Follow Command	3	2
Reading	1	0
Writing	1	0
Copying	1	0
Total Score	30	18

Initial laboratory investigations included CBC, which revealed anemia with Hb count of 11mg/dL; thyroid and kidney function tests were unremarkable. However, the liver function test revealed deranged enzyme levels (Table 3) with an R index of 2.30 which indicates mixed type of liver injury. Abdominal ultrasound revealed mild enlargement of liver with increased parenchymal echogenicity suggestive of fatty changes with no focal lesion [7].

**Table 3:** Liver function test report AST- Aspartate aminotransferase, SGOT- Serum glutamic oxaloacetic transaminase, ALT- Alanine aminotransferase, SGPT- Serum glutamic pyruvic transferase, GGT- Gamma glutamyl transpeptidase, ALP- Alkaline phosphatase, A:G- Albumin: Globulin.

Parameters	Result	Units	Reference range
AST (SGOT)	57	U/L	<40
ALT (SGPT)	64	U/L	<41
AST: ALT ratio	2.85		<1.00
GGT	13	U/L	<71
ALP	87	U/L	<128
Bilirubin Total	5.3	mg/dL	<1.10
Bilirubin Direct	3.9	mg/dL	<0.20
Bilirubin Indirect	1.4	mg/dL	<1.10
Total Protein	7	g/dL	6.40-8.30
Albumin	2.8	g/dL	3.97-4.94
A: G ratio	1.51		0.90-2.00

ECG was normal. The patient was transferred to the medicine ward for further workup, where, based on history, examination, and laboratory reports, a clinical diagnosis of pellagra was made, and the patient was put on nicotinamide supplementation (300mg/kg/day), balanced diet and vitamin supplements. In the following three days, there was a marked improvement in the skin lesions, thus confirming the diagnosis. He was further referred to the Department of Psychiatry for de-addiction counseling.

## Discussion

Pellagra, a disease considered to have died out years ago, continues to haunt underdeveloped and developing countries. Pellagra in a chronic alcoholic occurs due to a nutritionally deficient diet and the preferential absorption of alcohol in the gut. The initial cutaneous changes in pellagra resemble a sunburn. The skin is red, with large blebs or blisters that may accompany the erythema or may appear several days after its onset, marking the acute phase of the disease. The blisters may coalesce to form a bulla, which may rupture, causing bleeding and leading to dry brown scales with black crusts after a few weeks, a finding of chronic pellagra. In cases of severe deficiency, the skin becomes hard parchment-like with scales and deep pigmentation. The usually affected sites are the dorsal surface of hands, face, neck, arms, and feet. In our patient, the dorsal surfaces of the hands, legs, and arms were affected, and the skin was fissured. The lesions that extend up to the arm are called the glove or gauntlet form of pellagra. A well-demarcated broad band of lesions extending as a collar around the entire neck called- a Casal necklace is quite characteristic of the disease; it was, however, not seen in our patient [8]. Seborrheic dermatitis-like lesions on the nose are distinguished by erythema and sulfur flakes, which are yellow scales on follicular orifices. The following conditions must be kept in mind as differential diagnoses for skin manifestations (Table 4).

**Table 4:** Differential diagnosis of pellagra

Condition/disorder	Skin lesions
Photodermatitis	Patchy erythema with pruritus over the sun exposed areas
Dermatitis solaris	Exposed skin develops erythema and tenderness followed by blisters
Eczema	Dry cracked skin with pruritus and crusting
Pemphigus vulgaris	Painful blisters and erosions on the skin as well as mucous membrane
Lupus erythematosus	Malar rash, discoid rash over the face, scalp or elsewhere
Porphyria cutanea tarda	Blisters and erosions on the sun exposed areas followed by scarring

The symptoms of niacin deficiency manifest first in tissues with high energy turnover rates, like the gastrointestinal tract and the brain. This explains the early gastrointestinal involvement in the disease [9]. Dyspepsia, a burning sensation in the throat, constipation, and recurring diarrhea are the first signs of gastrointestinal abnormalities in pellagra, and these symptoms were present in our patient for the past three months. Inflammation of the intestinal mucosa due to an underlying disorder like Hartnup’s disease causes a decrease in the absorption of niacin and therefore such patients become more prone to pellagra. If left untreated, more severe symptoms could manifest like nausea, diarrhea, intestinal hemorrhage, stomach pain, and bleeding. Clinically, pellagra is characterized by the symptoms of burning (or scalded) sensation in the mouth and tongue, anorexia, burning in the anal region and urethra or vagina; various nervous and mental manifestations; emaciation, cachexia, and varying degrees of anemia [10].

The oral mucosa may develop ulcerations, red tongue, stomatitis, cheilitis, and red bald tongue. It may have an impact on the perianal and vaginal mucosa. Later stages may include insomnia, apathy, dementia, encephalopathic condition, and coma. The neurological symptoms of pellagra appear to be caused by a concomitant deficiency of vitamin B3- the dominant effector followed by vitamin B2 (riboflavin), B1 (thiamine), B12 (cobalamin), B6 (pyridoxine) and some nutrients like iron and zinc. However, a cause explaining their concomitant occurrence has not been found yet. A retrospective study conducted in India found a 47% prevalence of vitamin B12 deficiency in the North Indian population. Cobalamin deficiency has emerged as one of the most common nutritional problems in India and thus most cases of pellagra are found to be deficient in vitamin B12 already [11]. A variety of psychiatric symptoms may also be evident like headache, vertigo, tremors, weakness, myoclonus, seizures, and many others. Postmortem studies have shown chromatolysis of neurons, predominantly in the brainstem and cerebellum, but the exact cause is yet to be discovered. Nuclei of cranial nerves, and posterior horn cells in the spinal cord may also get affected giving rise to symptoms of peripheral neuropathy. Myelin, blood vessels are not affected [12]. Urocanic acid, which serves as an ultraviolet filter, is deficient, which results in photosensitive skin lesions. For cellular energy transfer reactions to continue in a patient of pellagra, NAD and NADP levels are insufficient. Two pathways may result in skin abnormalities when these cofactors are deficient: (i) a reduced ability to heal UV damage to the epidermis and (ii) a reduction in energy transfer to skin cells that replace themselves quickly. Through the accompanying malnutrition that reduces the availability and hinders absorption of niacin, its precursors, and other vitamins and nutrients, alcohol can cause pellagra. Additionally, alcohol’s inherent effects may amplify dietary deficit symptoms. Alcohol-specific side effects may include altered haem production, altered glutamate, and gamma amino butyric acid (GABA) neuronal activity, impaired tryptophan to niacin conversion, induction of zinc insufficiency, and metabolic implications of alcohol metabolism [8]. Pellagra is frequently identified by its typical skin lesions, sensitivity to niacin, improvement of neurological symptoms within days, and improvement of skin lesions over a week [13].

## Conclusions

We conclude that pellagra, although considered to have been eradicated from the developed world, sporadic cases continue to occur. Being a nutritional disorder, it is often seen in malnourished and alcoholic individuals or people who have an underlying disease. Seldom seen in children, a resurgence of its cases may occur due to poor and worsening eating habits, making children vulnerable. Pellagra, as a disease, can present with a myriad of symptoms, but the most distinguished are the dermatological manifestations seen. The characteristic of Casal’s necklace is almost confirmatory of the disease, but it may not always be present. Therefore, a knowledge of all the different symptoms and presentations is of utmost essential. While treating a case of niacin deficiency, one must also rule out deficiency of other vitamins and minerals. Most cases are deficient in riboflavin, cobalamin, and zinc in addition to niacin. Its association with dietary habits is an important factor to be kept in mind. Hence, practitioners must keep pellagra as a differential diagnosis when working in a vicinity where cases of pellagra may develop due to any of the risk factors and thus try to prevent the fourth “D”-death.
